# Table-Based Text Parsing Methods for Polymer Science: A Comparative Study

**DOI:** 10.1021/acsomega.6c05295

**Published:** 2026-09-04

**Authors:** Rian J. Howe, Jacqueline M. Cole

**Affiliations:** Ray Dolby Centre, Cavendish Laboratory, Department of Physics, J.J. Thomson Avenue, Cambridge CB3 0US, U.K.

## Abstract

Polymer informatics has emerged as an area of interest in the search for more sustainable plastics. Yet, there is a dearth in large, high-quality, polymer-based materials databases that are open-source for the polymer research community. Efforts to curate such databases involve autogenerating large experimental materials databases by mining chemical data from scientific literature, with a specific focus on mining tabular data as these are particularly rich sources of polymeric information. This study compares the performance of two tools in extracting large volumes of tabular data about polymer names and their glass-transition, melting and decomposition temperatures: a table-extraction tool that employs a downstream neural network to resolve polymer names from the table fields, and the table-mining part of the “chemistry-aware” natural-language-processing tool, ChemDataExtractor. We find that both methods afford high precision. The recall of the former is boosted by preserving some of the implicit structure of the source tables, while the latter offers a far wider scope of knowledge extraction from the literature on polymer science.

## Graphical abstract

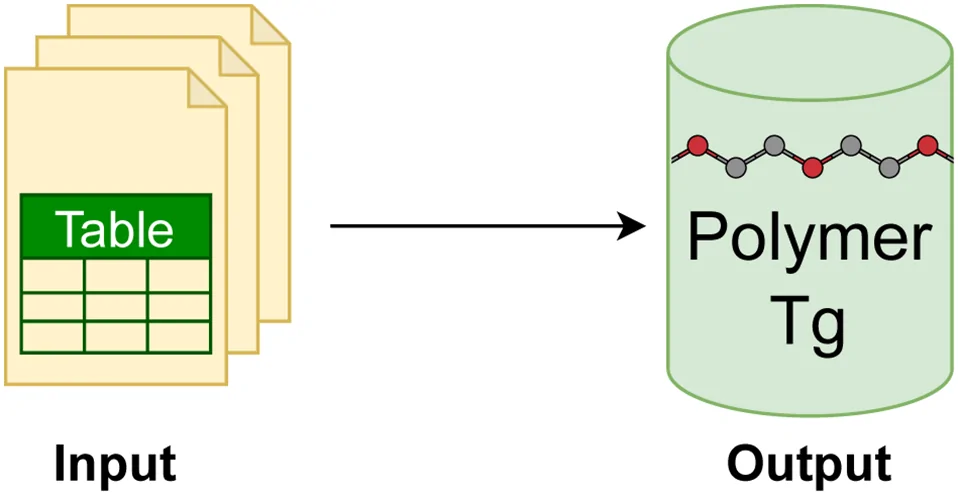

## Introduction

Polymers in the form of plastics represent one of the most widely used, but also controversial, materials in our modern society. Significant concerns have arisen about the sustainability of our insatiable demand for these materials, with over 430 million tonnes being produced worldwide each year, of which two-thirds are designed for short use and thus become waste.^1^ Reducing plastic waste has become a significant aim for both the United Nations^2^ and the European Union,^3^ highlighting it as a key issue worldwide. Tackling this ongoing problem will require a significant shift in the way that we create and consume polymers. One possible way forward involves adopting recent innovations in data-driven methods that provide a modern approach to discovering new, more sustainable materials. The potential for polymer informatics is vast, with an incredible number of polymer combinations being possible;^4–6^ thus affording myriad degrees-of-freedom in polymer design, the vast majority of which is yet to be explored.

Projects such as the Materials Genome Initiative (MGI)^7^ have helped to stimulate increased interest in and demand for data-driven materials discovery. A key aim of the MGI is to develop novel materials by harnessing and deploying data in the materials-science arena. Experimental research and development (R&D) efforts to produce new materials are very time and labor intensive; while a data-driven approach provides a more targeted method using data-science techniques to predict new candidate materials for a given functional application. Fundamental to this data-driven approach is the existence of large-scale, coherent materials data sets which provide the foundation for further materials development. The value of the insights which can be obtained from such data sets is highly dependent on their data quality; so it is imperative that they are accurate and unbiased.^8^ Moreover, materials databases which cover a diverse set of chemical materials and a wide range of properties are needed to unlock the full potential of the materials genome.^9–12^

Existing materials databases, with properties generated through computation using *ab initio* methods, can be sizable, but they can lack property diversity since only a handful of properties tend to be calculated in this way.^13^ Moreover, computationally generated materials databases tend to be highly idealized since calculations are often made on isolated molecules which typically behave very differently when placed in a real-world material application. This is especially true for polymers, whose relatively large molecular size means that typical electronic-structure calculations and molecular-dynamics simulations take days to compute for a single polymer ensemble. Recent studies in other domains^8^ have called into question the accuracy of data sets based on density functional theory (DFT), thereby challenging if it is appropriate to use them for material-property prediction models.

Manually curated databases that provide both computational and experimental data have been collected from peer-reviewed sources. One such example exists in the polymer domain: the PolyInfo database (https://polymer.nims.go.jp/en/),^14^ which is a manually curated database containing 470,000 data points including 61,000 values of glass transition temperature, *T*_*g*_ values. Crucially, this database is not available for public use, so cannot be used for open research in polymer informatics.

Polymer informatics has already been used to predict Tg values;^10,11,15–21^ yet, these studies have almost exclusively used computational data sources since they are available in readily prepared data sets. A large publicly available example of a polymer prediction platform is the Polymer genome project (https://www.polymergenome.org/),^22–24^ which predicts polymer properties from user inputted SMILES strings. The underpinning machine-learning (ML) models for these predictions were trained using data for 854 polymers from both computational (DFT) and experimental sources, which demonstrates the potential for property prediction from even a small selection of seed data.

Naturally, much larger data sets will be required to expand the property prediction to an order of millions of possible polymer molecules^4^ and to allow for a comprehensive polymer-design pipeline. Meanwhile, developments in data representation of polymers, that facilitate high-throughput studies on polymer structures for informatics are a demonstration of interest for data-driven methods that can afford polymer property predictions; a poignant example being BigSMILES.^25,26^

Most large-scale experimental materials databases have been curated by hand, often requiring several working lifetimes of human effort to produce; as such, those that exist required too much of an investment to justify making them publicly available for open research. Machine-based text-mining technologies offer an automated alternative, allowing for not only a much faster speed of data curation, but also a scalable approach, which is important in a world of exponentially increasing paper and data publications. Such data-mining methods have already been employed in fields where data can be found in large amounts of publicly available text, such as law,^27^ finance^28^ and the biomedical sciences.^29^ The field of materials science has seen a slower uptake of these techniques owing to the lack of standardization of chemical naming and property descriptions within publications and data sources in the field, which present major hurdles for data acquisition. In particular, difficulties in chemical named entity recognition (CNER) have been a bottleneck to progress. The “chemistry aware” natural-language-processing (NLP) software, ChemDataExtractor^30–33^ was developed to overcome these challenges. Thereby, ChemDataExtractor offers a dedicated toolkit for the data mining of chemical names and their cognate properties. This software has already been used to autogenerate many large-scale experimental databases that span fields such as thermoelectrics,^34^ batteries,^35^ alloys,^36^ semiconductors,^37^ photovoltaics^38^ and optical properties.^39^ The main source of data for these studies has been unstructured sentence-based scientific text from academic text. ChemDataExtractor has also been used to mine glass-transition temperatures^40^ of polymers on a very small scale (212 data records). More recently, ChemDataExtractor has been used to mine data from table-based sources of optical properties^41^ and alloys^42^ on a large scale, demonstrating that it can be deployed for this purpose effectively.

Progress has been made with methods to extract polymer names and properties from abstracts and paragraphs obtained from scientific articles at a large scale,^43,44^ but such progress has yet to be achieved on data contained within tables.

Polymer information appears to be predominantly found in tables within scientifc papers which, in contrast to sentence-based text, provides more implicit structure to the source data. So, one might presuppose that it could be more efficient to extract polymer information from tabular data. Yet, the lack of standardized table arrangements and polymer nomenclature mean that considerable data-extraction challenges are still present. One study, by *Oka et al.*,^45^ is of particular interest as it focuses on the table-based data extraction of polymer-based information. Their study adopted a different data-extraction approach to ChemDataExtractor.^30–33^ As such, we undertook a comparative study to understand the relative benefits and shortfalls of each method. This serves the broader goal of developing a robust set of table-based data-mining protocols for the field of polymer science.

## Methods

The principal steps involved in both types of information-extraction tasks in this comparative study are illustrated in Figure 1, and can be broadly split into two stages. First, table processing: a table is identified within its document file, and the table data are extracted in a format ready for feeding into an NLP pipeline. Second, property extraction: NLP interrogates the table data to identify and extract four property elements: Chemical name, Property specifier, Property value, Property unit. These elements are then resolved with each set of four forming a single record in the data set.

**Figure 1:**
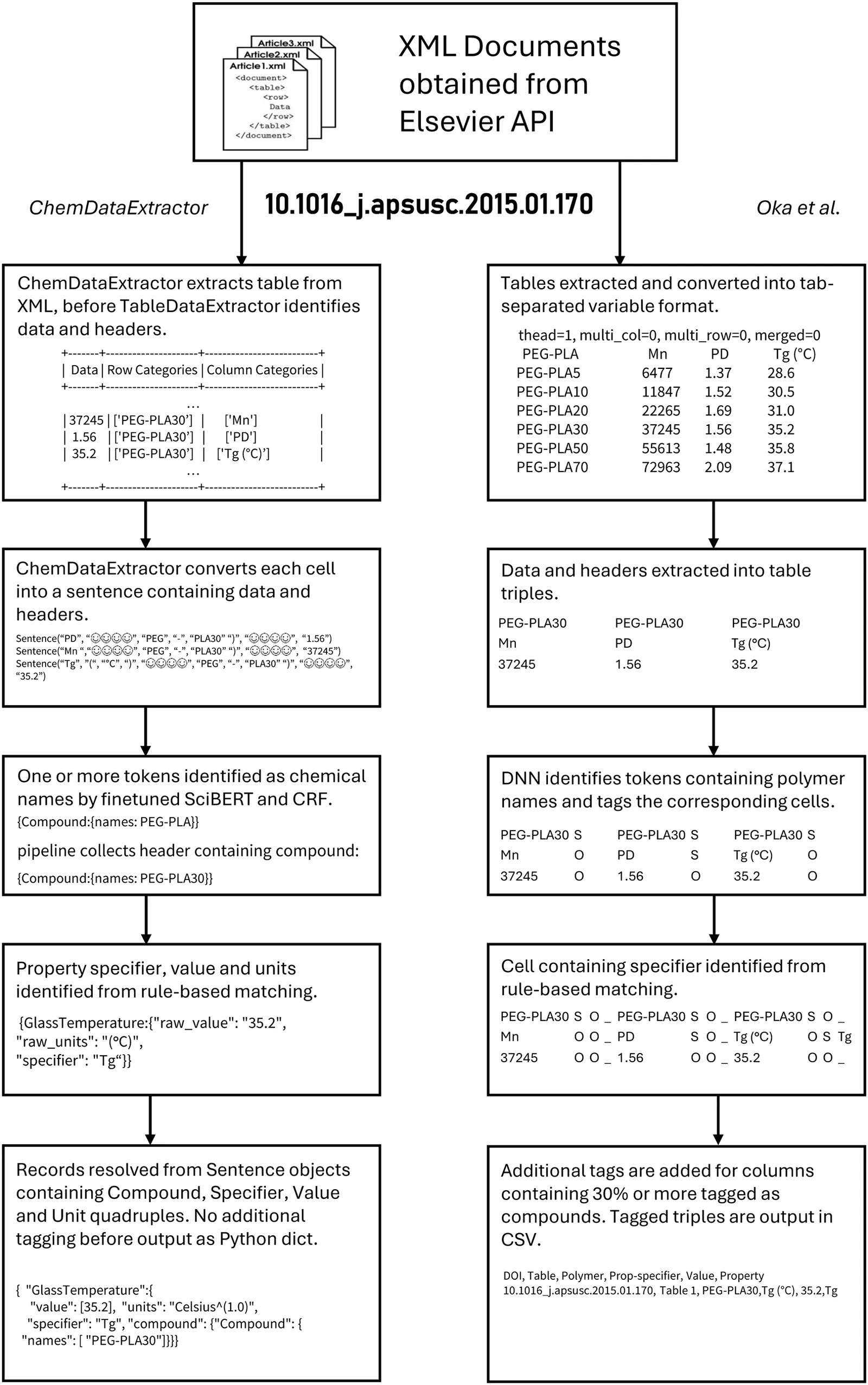
A comparison of the data-mining pipelines employed by *Oka et al.* ^45^ and ChemDataExtractor v2.4.^30–33^ Isolatory values of a case study from *Yao et al.* ^46^ were extracted and presented in a reformatted context for illustrative purposes alone. XML is eXtended Markup Language; API is application programming interface; CRF is conditional random field; DNN is deep neural network; CSV is comma separated value.

Both methods used very similar rule-based parsing methods to identify the property and value. The limiting factor in the property-extraction process was the chemical-named-entity recognition (CNER) step, where both methods took very different approaches.

For the purposes of evaluation in this work, both methods processed the same data set that was employed by *Oka et al*.^45^ This data set comprised a set of 18,602 full-text articles from the Elsevier publishing house. These articles were sourced in this study via the Elsevier Application Programming Interface (API) which secured them in extensible markup language (XML), which is a plain-text format allowing for machine processing the article text. Both methods are capable of interpreting the XML documents provided by Elsevier, and thus are able to identify table information within the documents obtained.

### ChemDataExtractor

The “chemistry-aware” text-mining tool, ChemDataExtractor version 2.4,^30–33^ was used in this work, whose CNER step is facilitated by the transformer model SciBERT, which had been pretrained on scientific text,^47^ and which has been fine-tuned for CNER utility. The table-processing method used by ChemDataExtractor version 2.4 ^30–33^ (Figure 1, left) first decomposes a table into its data cells which are tagged by their corresponding row and column headers. These data are then combined to form an artificial sentence, and passed into the property-extraction process.

The precision and recall of the compound names identified in the tables by ChemDataExtractor^30–33^ were improved for polymer science in this study, by making two modifications to its standard NLP pipeline. First, a dictionary of polymer names and abbreviations collected from the CROW polymer database^48^ were added for the CNER steps, to facilitate identification of common polymer names or abbreviations that may be missed by the SciBERT-based CNER model.^47^ Second, a modification was made whereby, when a compound name was retrieved, the raw cell text containing the full compound details was stored with the record; this allowed for the full chemical details to be captured, even when only a partial match of the compound information has been realized by the SciBERT-based CNER model *e.g.*, if the cell contains ″PFDA10-*b*-PCL20″ and the CNER retrieves ″PFDA″ as the compound name, then ″PFDA-*b*-PCL20″ is added to the compound record. The codebases for these two modifications to ChemDataExtractor are provided in a public repository, details of which can be found in the Data Availability section.

User-defined rules dictate the search for property specifiers and the measurement type (in this case temperature), and then predefined rules identify the value and the units. ChemDataExtractor is already configured to look for Celsius/Kelvin/Fahrenheit, but for more complex properties custom units can be added. The distinct identification of the specifier and the units separately differs from *Oka et al*. A record is formed if all four of the compound, specifier, unit and value are found in a single sentence created from a table cell.

### The Approach by *Oka et al.*

Each step of the pipeline developed by *Oka et al.* ^45^ (Figure 1, right) is applied to all the documents in their target data set before advancing to the next step; this differs significantly to ChemDataExtractor which applies the full pipeline to each document one by one.

The process by *Oka et al.* ^45^ first separates the tables from the remaining document text and converts them into tab-separated variable (tsv) format. All the extracted table information is then saved into a file for each journal article. The next step reads these tsv files, and forms table “triples” from the data cells with the corresponding row and column header cells. These triples are then combined in a single file for multiple articles. An additional file records the position of the data cell within the table and journal from which it was extracted.

Their CNER step is then undertaken which employs a deep neural network (DNN) architecture from Dos Santos and Zadrozny,^49^ that performs part-of-speech (POS) tagging using character-level embeddings in addition to word embeddings. This DNN model was trained on 588 articles that had been annotated using a rule-based search for single tokens containing “P” or “poly”, alongside stop words and some common exceptions. This DNN model was applied to the tokens in the table triples, and any cells that contain recognized tokens were tagged. The method employs an additional interdependency step, which follows the logic: if more than 30% of cells in a column are tagged to contain chemical named entities, all of the cells in that column are tagged. Although this interdependency step is simple, it allows for the algorithm to effectively use information regarding the table structure in a way that ChemDataExtractor does not.

Since the pipeline employed by *Oka et al.* relies on tagging table cells as whole entities, the cell containing property specifier and the pipeline assumes that the unit is contained inside. This of course adds a required layer of postprocessing to remove the unit, but crucially it also opens up the possibility of errors where the pipeline tags cells which do not contain temperatures, for example TGA is a common abbreviation for thermogravimetric analysis which is a measurement technique. This stage of the pipeline uses string matching alongside allowlist and blocklist files for each property which have been supplied with the model published by *Oka et al*.

## Evaluation

We see from Table 1 that the numbers of tables obtained in both pipelines are consistent with what is expected using a simple Pythonic method with *lxml*. Thus, we assume that the table identification parts of both pipelines are able to identify tables correctly.

**Table 1:** Comparing the Number of Tables Obtained from the Data Set by Each Method to a Simple Pythonic XML (Using the *lxml* Package) Extraction of the Table from the XML

Method	Number of articles	Containing tables	Number of tables
*lxml*	18,602	13,201	30,936
ChemDataExtractor	18,602	13,200	30,935
*Oka et al.*	18,602	13,201	30,936

The transition temperature data obtained from both methods described in this study are compared against each other and against corresponding data from the PolyInfo database that was sourced by *Oka et al.* ^45^ Figure 2 shows that the data extracted by both methods presented herein follow roughly the *T*_g_, *T*_*m*_, *T*_*d*_ distributions of the PolyInfo data set. This suggests that the data obtained by both methods compared herein are representative of those found in the academic literature and are not biased toward a particular group of polymer materials.

**Figure 2:**
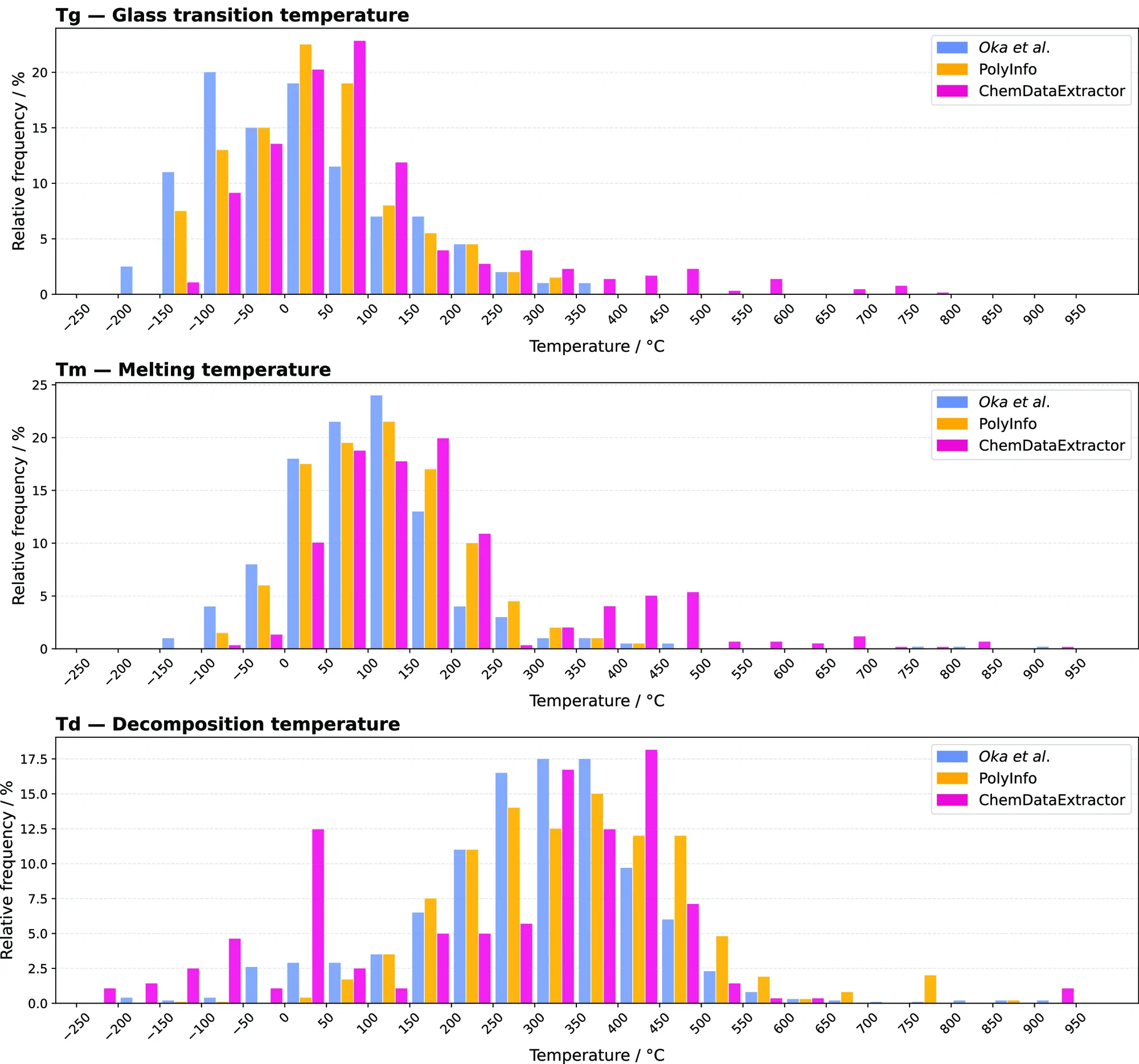
Histogram comparing data on glass-transition temperatures, that were obtained from processing tables using the methodology of *Oka et al.* and ChemDataExtractor, using a common corpus, with reference to the values contained in the PolyInfo database^45^ that were relayed by *Oka et al*. The three types of data distributions largely track each other within the ranges of high frequency data. ChemDataExtractor appears to capture more data at the extremities of these distributions, especially at the higher temperatures.

**Figure 3:**
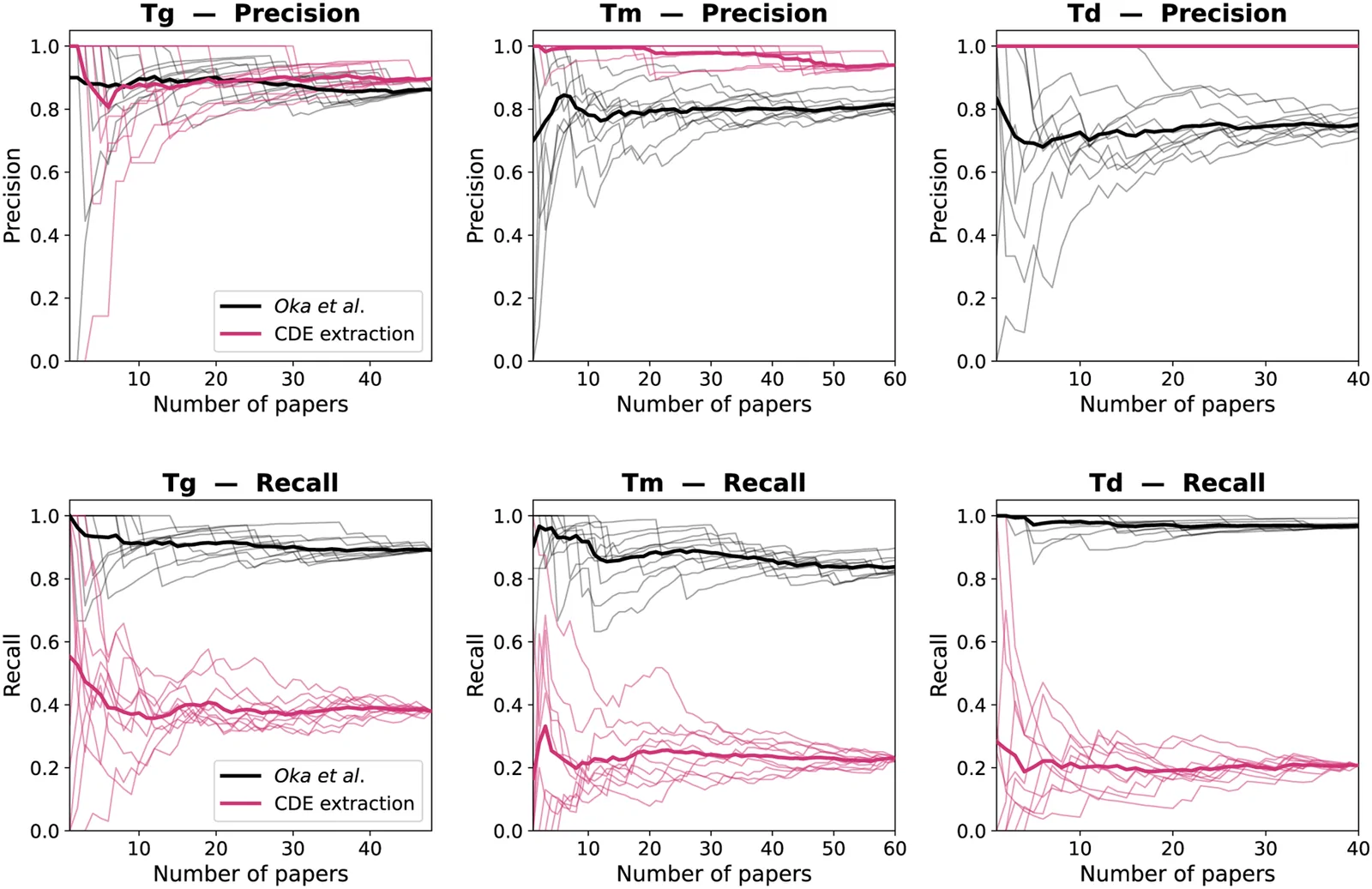
Cumulative recall and precision metrics calculated by the authors using the methodology of *Oka et al.* ^45^ and ChemDataExtractor^30–32^ (labeled CDE herein) for 10 random orders of the evaluation data set, with the mean values plotted in bold. Since the values converge, we can assume that they are representative of the whole data set.

*Oka et al.* ^45^ noted a deviation from this histogram for the −50 to 0°C *T*_g_ range, which is reproduced in this study. We also note large deviations from this histogram for the 0 to 50°C and 400 to 450°C *T*_*d*_ ranges for the data obtained using ChemDataExtractor. Further inspection reveals that several of the values between 0 and 50°C are in fact lost percentage mass values at a given degradation temperature which explains the deviation.

The same evaluation data set was used as in the *Oka et al.* study^45^ to ensure consistency of 100 polymer journal articles, and 100 nonpolymer journal articles. The ground-truth data of *T*_g_, *T*_*m*_, *T*_*d*_ were manually extracted from the tables inside these articles and then compared to the extracted data obtained from both methods. Since both methods output the table cell containing token(s) identified as compounds, our ground-truth compound data were the cell text. This meant partial matches of tokens were considered to be correct as long as they referenced back to the correct complete table cell text. Only records consisting of the correct compound, property and value were considered to be correct. Crucially, no errors were seen to result from table processing; rather, a vast majority of errors came from missed or misidentified compound cells.

1 $$\mathrm{Precision}=\frac{TP}{TP+FP},,\mathrm{Recall}=\frac{TP}{TP+FN},,F_{1}=\frac{2\times \mathrm{Precision}\times \mathrm{Recall}}{\mathrm{Precision}+\mathrm{Recall}}$$

## Discussion

The overall numbers of each property extracted by both methods shown in Table 2, We see that ChemDataExtractor obtains significantly fewer records than *Oka et al*. This is particularly true in the case of decomposition temperature, *T*_*d*_ where there are a range of property descriptions *e.g.*, *T*_*onset*_, *T*_10%_, *T*_*deg*_ corresponding to different definitions *i.e.*, onset of mass loss, 10% mass/volume lost, 50% mass/volume lost.

**Table 2:** Total Numbers of Each Property Extracted by Both *Oka et al.* and ChemDataExtractor

Method	*T*_g_	*T*_*m*_	*T*_*d*_
ChemDataExtractor	661	686	290
*Oka et al.*	2,181	1,526	2,316

Table 3 and Figure 3 compare the evaluation metrics obtained using eq 1, from which we see that both comparative methods achieve very similar precision values, with ChemDataExtractor^30–32^ affording a small improvement over that of *Oka et al*.^45^ The quality of the data output by both methods can therefore be considered to be similar, in the context of the data used in this study.

**Table 3:** Comparison of the Evaluation Metrics (Calculated Using eq 1) for Both Methods by *Oka et al.* ^45^ and ChemDataExtractor^30–32^ for the Three Transition Temperatures Extracted and Their Combined Set

Method	Property	Precision	Recall	F1 Score
*Oka et al.*	*T*_g_	0.862	0.891	0.876
	*T*_m_	0.810	0.844	0.826
	*T*_d_	0.757	0.972	0.851
	**Combined**	**0.809**	**0.892**	**0.848**
ChemDataExtractor	*T*_g_	0.897	0.380	0.534
	*T*_m_	0.941	0.226	0.365
	*T*_d_	1.000	0.207	0.344
	**Combined**	**0.931**	**0.271**	**0.420**

Given that the CNER step in ChemDataExtractor^30–32^ employs the SciBERT model that was pretrained using annotated text prose from sentences from scientific articles, it cannot necessarily be assumed that it will be able to perform CNER effectively when applied to sentences which have been constructed from table cells. These constructed cells lack the context supplied by the additional words in a free text sentence, and hence we would expect the SciBERT-tagger to be significantly more sensitive when processing these sentences. An extreme case of this sensitivity appeared in one case of the evaluation: two sentences were obtained, one containing a melting point, one containing a glass transition temperature, for the same compound, from the same table and only the compound in the melting point sentence was tagged. This sensitivity to the lack of words providing context in the generated sentence would explain the very low recall scores obtained by ChemDataExtractor in this study, especially when compared to previous works using ChemDataExtractor,^34–39^ which have achieved much better recall when processing free text sentences.

Some interdependency-resolution issues reported occurred with both methods, namely labels and numbers being used to represent chemicals. These issues notwithstanding, the accompaniment of metadata with the chemical property data is especially important for describing a polymer with a data representation.

In contrast, a big difference is observed when comparing the recall values of both methods (shown in Table 3) and the total numbers of each property obtained (shown in Table 2), whereby the method by *Oka et al.* ^45^ captures a larger amount of the target data that are held within the corpus used in this study. However, the corpus used for this study consists of 18,602 “generic” polymer papers *i.e.*, papers that are not specific to the target property of this study; as such, only 1,600 articles contained the target phrase “glass transition”, and the target-property records collected were only found in approximately 300 of those papers. Therefore, if this study was to be scaled up to a larger or more target-property-specific corpus, we would still expect to obtain a useful amount of records with both methods.

The corpus chosen by *Oka et al.* for evaluation consisted of 100 polymer journal articles and 100 nonpolymer journal articles. The resulting evaluation published by *Oka et al.* shows that their pipeline returned data for 157 of those articles. This means that 79% of their evaluation data set returned data. In comparison, 281 articles out of the 18,600 in the full corpus returned results using the pipeline from *Oka et al.*, only 1.5% of the original corpus. This large discrepancy in the sparsity of data in the evaluation data set to the corpus means that the evaluation data set is not reflective of the full corpus. Furthermore, this suggests that the data set has been biased toward articles that return results using the *Oka et al.* pipeline and will likely produce better evaluation metrics for the *Oka et al.* pipeline than an alternative such as ChemDataExtractor. This is especially significant given the large difference in recall between the two methods when evaluated in this study.

Table 4 illustrates the vast difference in processing time, with ChemDataExtractor taking 3.5 min on average per article. It was deployed on the ALCF Polaris supercomputer for this reason, making use of the 32-core CPU to process 32 process papers simultaneously. In contrast, *Oka et al.* method is vastly quicker, taking only 2.4 s per article on average when deployed in this study.

**Table 4:** Comparison of the Hardware and Processing Times Used to Extract the Data in This Study^a^

Method	Running time	Hardware	Average/article
ChemDataExtractor	35.5 h	AMD EPYC Milan 7543P 32-core	3.5 min
*Oka et al.*	12.5 h	Intel i5-8500 6-core CPU	2.4 s

a ChemDataExtractor processed 32 articles simultaneously .

Interestingly, the major bottleneck in the *Oka et al.* pipeline was observed to be the *tsv* table extraction process, taking on average 2.1 s/article. This part of the pipeline is substantially quicker in ChemDataExtractor, taking around 0.25 s/article. The *lxml* tree used as the ground-truth for table identification in Table 1 shows that both methods produce an almost identical number of tables and are consistent with the expected value. Additionally, in the evaluation data sets obtained for both methods, all of the tables were parsed correctly and no downstream errors were seen due to table parsing problems.

The innate scope of the pipeline employed by *Oka et al.* ^45^ is considerably narrower than that of ChemDataExtractor,^30–32^ with the CNER employed by *Oka et al.* ^45^ being only applicable to polymer names. In contrast, ChemDataExtractor^30–32^ not only allows for the CNER of materials outside of the polymer space, but it can easily be employed to extract additional properties and, in particular, group properties together for individual chemical records where needed. Furthermore, ChemDataExtractor is not limited to XML and can process documents (including tables) from HTML and plain text formats. Given that PDF to markdown conversion is possible with tools such as Docling, this also extends the domain of ChemDataExtractor to table data extraction from PDF documents. In other words, ChemDataExtractor is far more extensible given its generic data-mining approach.

During this study, we observed that tables are common in the polymer domain and data-rich with multiple properties and compounds; as such, they could provide a very promising source of data for this field of chemistry; yet, inconsistencies in chemical naming and the structures of tables themselves present challenges to overcome. The method by *Oka et al.* ^45^ preserves some of the table structure by tagging cells instead of tokens and, in particular, tagging entire columns if 30% or more of the cells are tagged as containing polymer mentions. This simple step accounts for a large amount of the recall in the evaluation results of *Oka et al.* ^45^ and hence it is crucial in its performance. Indeed, when removing this step, we obtain 1,678/1,199/1,761 records for *T*_*g*_/*T*_*m*_/*T*_*d*_, respectively, compared to the 2,181/1,526/2,316 with the rule included, meaning that this step alone accounts for a 27% increase in recall. Since the ChemDataExtractor^30–32^ method converts table structures into sentences, it does not preserve the structure of the table. While this study modified the ChemDataExtractor^30–32^ pipeline to collect the table cells containing tokens that have been tagged by the CNER instead of the tagged token(s), the loss of the table structure happens early in the pipeline during the table-parsing stage; hence, adding a feature similar to the one employed by the method of *Oka et al.* would require the entire pipeline of ChemDataExtractor^30–32^ to be reconfigured.

## Conclusions

This study has demonstrated that ChemDataExtractor v2.4 ^30–32^ and the method by *Oka et al.* ^45^ can both mine tabular data from scientific documents about polymer names and their properties, with a comparatively high level of precision. This is despite our finding that tables rich in polymer data within the academic literature lack standardization, both in terms of the formatting of table structures and data contained within tables. This goes against the naïve expectation that data extraction from tables might be easier than that of text prose because tables are more structured sources of data than sentences; and are much denser in data content. Given that the information contained with the tables is significant, future methods to extract structure–property relationships about polymers should not overlook the value of table content despite these standardization issues. Indeed, certain “quick fixes” can circumvent these issues; as illustrated by *Oka et al* ^45^ who poignantly demonstrated that the preservation of just some of the table structure affords a dramatic increase in recall.

Ideally, the extraction of polymer data from both tables and text prose would be most beneficial. ChemDataExtractor v2 ^30–32^ was designed to accommodate this wider scope, at scale, using its modular pipeline with multiple parsers, *cf*. its AutoTableParser and AutoTemplateParser. Moreover, a large language model (LM) is embedded into the CNER step of ChemDataExtractor v2.4, used in this work; and in all previous ChemDataExtractor versions since v2.1. Given the increasing appetite for LM-based problem-solving, one could widen the LM-based aspects of ChemDataExtractor. Indeed, others have already created an LM called PolyBERT^5^ that has been pretrained on a polymer corpus. Looking ahead, one could go further to develop a multimodal model that feeds an LM with textual, tabular, imaging, and graphical data, in parallel streams. Once fine-tuned on data extraction, these different types of data could be acquired concurrently, and analyzed cooperatively by using the correlations between them.

## Data Availability

The code for two modifications of ChemDataExtractor v2.4 ^30–33^ undertaken for this study is contained in the files *cem_factory.py*, *table.py*, *model.py,* and *abbrev.py,* which are available online.^50^ Minor modifications to the source code published by *Oka et al.* ^45^ were made purely to update the software and ensure it ran on the desktop computer used; these are available online.
